# MiR‐362 suppresses cervical cancer progression via directly targeting BAP31 and activating TGFβ/Smad pathway

**DOI:** 10.1002/cam4.3601

**Published:** 2020-11-19

**Authors:** Shuya Yang, Yuanjie Sun, Dongbo Jiang, Jing Wang, Erle Dang, Zichao Li, Jiayi Zhou, Yuchen Lu, Jingqi Shi, Liang Tao, Jun Wang, Boquan Jin, Lianhe Zheng, Kun Yang

**Affiliations:** ^1^ Department of Immunology The Fourth Military Medical University Xi’an Shaanxi People’s Republic of China; ^2^ Department of Dermatology Xijing Hospital The Fourth Military Medical University Xi’an Shaanxi People’s Republic of China; ^3^ School of Basic Medicine The Fourth Military Medical University Xi’an Shaanxi People’s Republic of China; ^4^ Department of Medical Microbiology and Parasitology The Fourth Military Medical University Xi’an Shaanxi People’s Republic of China; ^5^ Department of Orthopedics Tangdu Hospital The Fourth Military Medical University Xi’an Shaanxi People’s Republic of China

**Keywords:** anti‐oncomiR, BAP31, cervical cancer, miR‐362, TGFβ/Smad pathway

## Abstract

BAP31 (B‐cell receptor‐associated protein 31) is an important regulator of intracellular signal transduction and highly expressed in several cancer tissues or testicular tissues. Our previous study had revealed that elevated BAP31 plays a crucial role in the progress and metastasis of cervical cancer. Even so, the precise mechanism of abnormal BAP31 elevation in cervical cancer has not been fully elucidated. We revealed that the expression of BAP31 was mainly regulated by microRNA‐362 (miR‐362), which was markedly downregulated in cervical cancer tissues and negatively correlated with clinical tumor staging. Overexpression of miR‐362 inhibited cervical cancer cell proliferation and increased the proportion of apoptotic cells. Furthermore, miR‐362 reduced the tumor sizes and prolonged mice survival time in xenograft nude mice model. Finally, we demonstrated that the BAP31/SPTBN1 complex regulated tumor progression through the Smad 2/3 pathway under the control of miR‐362. Collectively, our findings demonstrated that miR‐362 could work as an anti‐oncomiR that inhibits proliferation and promotes apoptosis in cervical cancer cells via BAP31 and TGFβ/Smad pathway. Overexpression of miR‐362 might be a potential therapeutic strategy for cervical cancer.

## Graphical abstract

Through in vivo and in vitro experiments, our findings demonstrated that miR‐362 works as an anti‐oncomiR that inhibits proliferation and promotes apoptosis in cervical cancer cells via BAP31 and TGFβ/Smad pathway. Overexpression of miR‐362 might be a potential therapeutic strategy for cervical cancer.

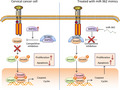

## 1 INTRODUCTION

Cervical cancer (CC) is the fourth most common woman malignancy that is diagnosed worldwide, with about 530,000 new patients and 270,000 deaths annually.^1^ About 85% of CC deaths worldwide occur in underdeveloped or developing countries, which is 18 times higher than that in rich countries.^2^ Still, the pathogenesis and treatment strategies of CC have not been well resolved.

Our research group previously discovered a new cancer/testis antigen BAP31 (B‐cell receptor‐associated protein 31),^3^ which is strongly expressed in several cancer or testicular tissues, especially in CC.^3^ In addition, BAP31 expression is positively correlated to clinical staging of CC.^3^, ^4^ Moreover, BAP31 regulates CC cell progression. The downregulation of BAP31 prevents the development of CC in vivo.^3^, ^4^ Taken together, BAP31 performs an essential role in the pathogenesis of CC and may be a potential target for therapy. However, the exact mechanism of abnormal BAP31 high expression in CC has not been fully elucidated.

MicroRNAs (miRNAs) are short noncoding RNAs. They mainly downregulate the gene expression posttranscriptionally and act as important parts to many physiological or pathological processes, including in CC cells.^5^, ^6^, ^7^ It has been found that miR‐362 acts as a tumor suppressor in several types of cancer. It directly downregulates the expression of E2F1, USF2, and PTPN1, and results in the disruption of cell cycle in colon cancer.^8^ MiR‐362 could also inhibit the development of breast cancer by reducing the expression of p130 Crk‐related substrates.^9^ Recent studies have found that miR‐362 is downregulated in CC and functions as a tumor suppressor miRNA.^10^, ^11^

In this study, we screened and demonstrated that miR‐362 has a strong regulatory effect on BAP31 expression. MiR‐362 overexpression inhibited CC cell proliferation and increased the proportion of apoptotic cells. In nude mice, miR‐362 reduced the tumor sizes and prolonged mice survival time. Upon further research, we demonstrated that the BAP31/SPTBN1 complex regulates the tumor progression through the TGFβ/Smad pathway under the control of miR‐362. Overall, our findings establish miR‐362 inhibits proliferation and promotes apoptosis via BAP31 and TGFβ/Smad pathway.

## 2 MATERIALS AND METHODS

### 2.1 Clinical tissue sample collection

Clinical tissue samples (including CC and adjacent tissues) were attained from patients with CC in our hospital, with clinically and pathologically confirmed. All patients were informed and agreed to all items. This study was confirmed by the ethics committee of our hospital.

### 2.2 Cell culture and transfection

HeLa and 293 T cell lines (Genechem Co., Ltd.) were cultured in DMEM (Gibco) and SiHa cell lines in MEM (Gibco), both with 10% fetal bovine serum at 37°C in 5% CO_2_. The miRNA mimics, inhibitor, negative control, BAP31 siRNA, and BAP31 plasmid were transiently transfected into cancer cells using the Lipofectamine 3000 reagent. The cells were treated for 24 h, then cultured in drug‐free medium. The miR‐362 mimics, inhibitor, negative control, BAP31 siRNA, and BAP31 plasmid were synthesized by GenePharma and Sangon Biotech. The sequences are in Table S1.

### 2.3 Flow cytometry assay

For cell cycle test, the transfected cells were first collected at 48 h after cell transfection, then fixed in 70% ethanol at 4°C overnight, and finally stained with PI (propidium iodide). For the apoptosis test, cells are first digested at 48 h after cell transfection and then, washed with PBS. According to the instructions (Biolegend), we stain the cells with PI and annexin V‐fluorescein isothiocyanate. Novoexpress software (ACEA Biosciences) was used to analyze the data.

### 2.4 Immunoblotting

The Wes robot (ProteinSimple) was used to perform a simple immunoblot analysis using a Master Kit with the standard pack, wes prefilled plate, secondary antibodies, and buffers (12–230 kDa and 66–440 kDa). The protein was collected at 48 h after cell transfection or from wild‐type cells. We used Compass software (ProteinSimple, version 3.1.7) to program the Wes robot and demonstrate (and quantify) Western immunoblots. The antibodies used for immunoblotting are from CST (Cell Signaling Technology) for caspases, Smad pathway proteins, and SPTBN1, Abcam (Abcam) for BAP31 and cyclin family proteins, and Proteintech (Proteintech) for Actin.

### 2.5 Luciferase reporter assay

We used the GP‐miRGLO reporter vector (GenePharma) to synthesize the human BAP31 3'‐UTR reporter plasmid, including the putative binding sequence of miR‐362 (wild type, WT) and mutant sequence (mutant, MU). The reporter plasmid was verified by sequencing. We transfected HeLa and SiHa cells with reporter plasmids and negative control (NC)/miR‐362 mimics. After 48 h, we tested the luciferase activity using Dual‐Luciferase Reporter Assay System (Promega). Renilla luciferase serves as the internal transfection control.

### 2.6 Quantitative real‐time PCR analysis (qRT‐PCR)

Total RNA was collected from cells or tissues by the use of TRIzol (Takara) reagent from wild‐type cells or cells at 24 h after cell transfection. The qRT‐PCRs of miRNAs were performed using SYBR^®^ PrimeScript™ miRNA RT‐PCR Kit (TaKaRa Bio Group). U6 served as an internal control for miRNA analysis. We normalized all samples to internal controls and calculated fold change by quantitative relative method (2^−ΔΔCT^). The primers used for analysis are found in Table S2.

### 2.7 Bioinformatics analysis

The TCGA database and their corresponding clinical information were downloaded from https://xenabrowser.net/datapages/. The survival analyses of miR‐362 in cancers were performed with R language (Ribobio).

Gene microarray and the analyses for differential gene expression and KEGG (Kyoto Encyclopedia of Genes and Genomes) were performed with R language (Genechem).

### 2.8 Tumor xenografts

To generate tumor xenografts, HeLa cells were injected (1 × 10^6^ cells per site) into nude mice subcutaneously (6–8 weeks old). The mice were randomly grouped (4 mice/group for 3 weeks’ treatment and 5 mice/group for survival observation). The mice were then injected with miR‐362 mimics, NC, or BAP31 siRNA (10 μg per tumor per time) using vivo‐jetPEI delivery agent (Polyplus) in 5% glucose on postoperative day 14. The injections were repeated every 3 days. Tumor sizes were measured before every injection with calipers and computed by (large diameter) × (short diameter)^2^/2 (mm^3^). The animal research has been confirmed by the institutional review board.

### 2.9 Immunohistochemistry staining and immunofluorescence confocal microscopy

For Immunohistochemistry staining, we sliced the tissues into the thickness of about 4 µm. The specimens were dewaxed, antigen retrieval, and blocked in sequence. Incubate BAP31 antibody overnight at 4°C and secondary antibodies at room temperature for 1 h. DAB chromogen (Dako) was used for immunostaining. We digitized the immunohistochemistry results using the Nanozoomer Digital Pathology System (Hamamatsu). For immunofluorescence confocal microscopy, the mouse antibody of BAP31 is generated by our research group,^3^ and the rabbit antibody of SPTBN1 is purchased from Proteintech (Proteintech). The statistical results of immunofluorescence colocalization were provided by Genechem (Genechem).

### 2.10 Statistical analysis

All the experiments were repeated at least three times, and statistical analyses were conducted by the use of GraphPad Prism 7.0 (GraphPad Software). For experiments with more than two groups, the differences were compared by one‐way ANOVA and the Dunnett's test, with the control group as a reference. Student's *t*‐test was used for experiments with only two groups. Survival rates were analyzed by the Kaplan–Meier method and log‐rank *t* test. *p* < 0.05 was considered to be statistically significant.

## 3 RESULTS

### 3.1 miR‐362 downregulates BAP31 expression by directly targeting BAP31 3'‐UTR

**FIGURE 1:**
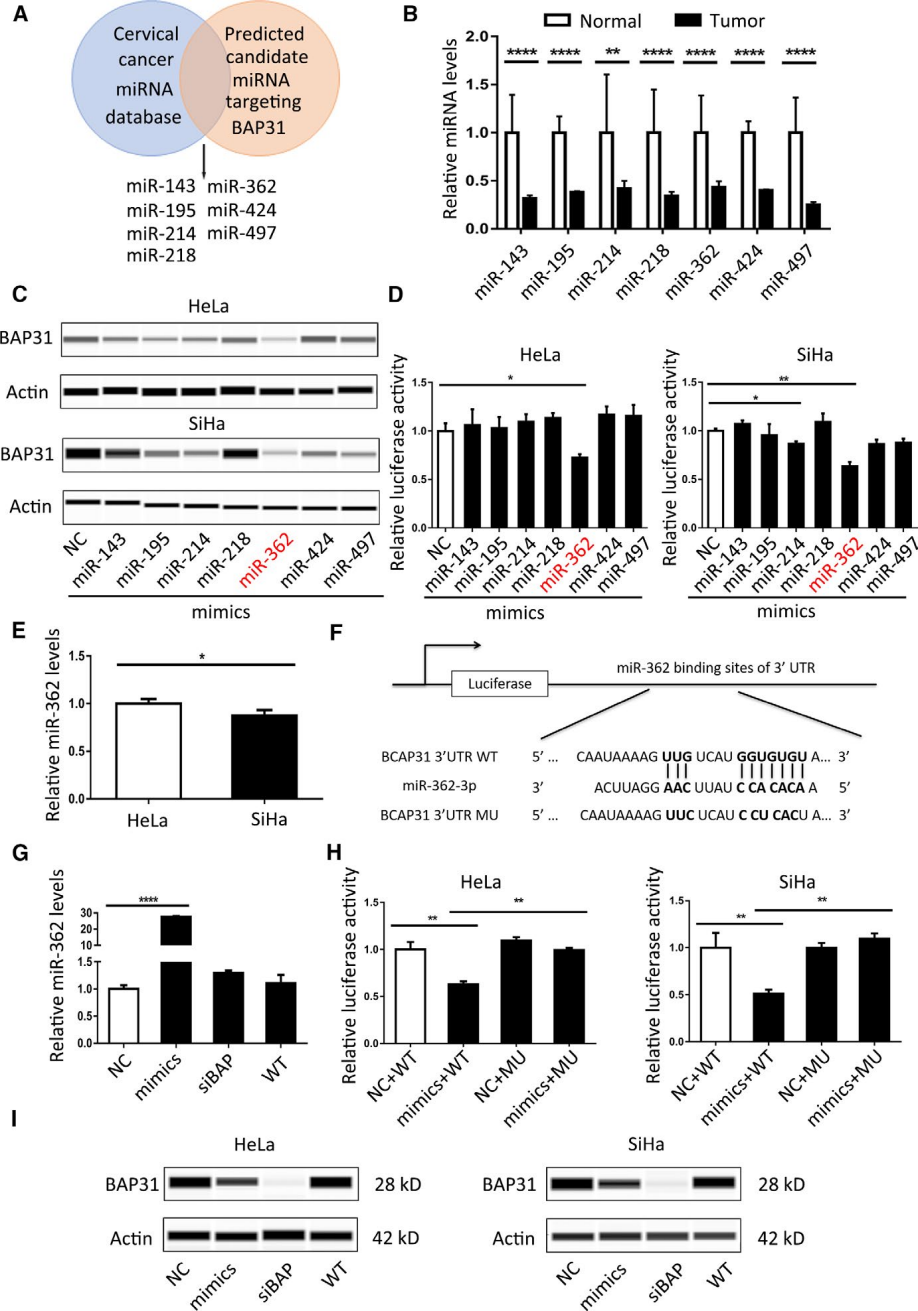
MiR‐362 downregulates BAP31 expression by directly targeting BAP31 3'‐UTR. (A) Venn diagram depicting potential miRNA candidates that may regulate BAP31 expression in cervical cancer. (B) qRT‐PCR results of miRNA expression levels in normal and cervical cancer tissues, 12 cases in normal group and 28 cases in tumor group. (C) Immunoblotting analyses for BAP31 protein levels following transfection of seven miRNA mimics into HeLa and SiHa cells. (D) Results of luciferase reporter assays in HeLa and SiHa cells co‐transfected with BAP31 WT (wild type) 3′‐UTR vectors and seven miRNA mimics, respectively, for 48 h. (E) qRT‐PCR result of miR‐362 expression in HeLa and SiHa cell line. (F) Diagram of BAP31 3'‐UTR WT and MU vectors for the putative target sequence of miR‐362. WT: wild type; MU: mutant type. (G) qRT‐PCR analyses for miR‐362 level following transfection of miR‐362 mimics, NC (negative control), and siBAP (BAP31 siRNA) into HeLa cells, U6 RNA was used as a control. (H) Results of luciferase reporter assays in HeLa and SiHa cells co‐transfected with BAP31 WT/MU 3′‐UTR vectors and miRNA mimics for 48 h. (I) Immunoblotting analyses for BAP31 protein levels following transfection of NC, miR‐362 mimics, and siBAP into HeLa and SiHa cells. Transfection with BAP31 siRNA (siBAP31) acted as a positive control. *p < 0.05; **p < 0.01; ****p < 0.0001. Data are represented as the mean ±SD of three independent experiments

In order to explain the posttranscriptional regulation mechanism of BAP31, we examined and identified the possible miRNAs that may induce BAP31 overexpression in CC. We predicted candidate miRNAs targeting BAP31 3′‐UTR (3′‐untranslated region) in TargetScan and microRNA (microRNA.org) databases. Then, we filtered out miRNAs that are downregulated in CC from published databases. Based on these analyses, we got miR‐143, miR‐195, miR‐214, miR‐218, miR‐362, miR‐424, and miR‐497 as candidates for regulating BAP31 in CC (Figure 1A). For confirming the expressions of the above miRNAs, we tested them in CC tissues (*n* = 28) and adjacent normal tissues (*n* = 12). The result of qRT‐PCR analysis showed that the above miRNAs were all under expressed in CC tissues, compared with that in adjacent normal tissues (Figure 1B).

To find the miRNA that effectively targets BAP31, we transiently transfected the miRNA mimics into HeLa or SiHa cell lines. MiR‐362 had a significant inhibitory effect on the expression of BAP31 after transient transfection compared with other miRNAs, as revealed by immunoblotting (Figure 1C). After co‐transfection of the vector containing BAP31 3′‐UTR sequence and the seven miRNA mimics, respectively, into HeLa or SiHa cells and test in the dual‐luciferase reporter system, we found miR‐362 had the strongest ability to bind to BAP31 3′‐UTR (Figure 1D). In addition, we tested the expression level of miR‐362 in CC cell lines, and the results showed that miR‐362 was lower in SiHa compared with that in HeLa cell line (Figure 1E).

To verify BAP31 as a miR‐362 target, we conducted a luciferase‐binding assay. According to the seed sequence in miR‐362 and the matching sequence in BAP31 3′‐UTR from TargetScan and microRNA (microRNA.org) databases, we constructed BAP31 3′‐UTR wild‐type and mutated vectors (Figure 1F). The qRT‐PCR results showed that miR‐362 mimics transfection could increase the expression level of miR‐362 significantly, and siBAP (BAP31 siRNA) does not affect miR‐362 expression, compared with NC and wild‐type group (Figure 1G). Luciferase reporter system results demonstrated that miR‐362 mimics could significantly reduce the fluorescent activity, and this effect could be abrogated the by mutant vector (Figure 1H). Along with miR‐362 levels rising in HeLa and SiHa cells through transiently transfected with miR‐362 mimics, the protein expression level of BAP31 decreased (Figure 1I). BAP31 protein expression level increased after transfection of miR‐362 inhibitor in 293 T cells (Figure S2). Together, these results indicated that miR‐362 downregulates BAP31 expression by directly targeting BAP31 3′‐UTR.

### 3.2 Reduced miR‐362 levels in cervical cancer associated with BAP31 protein overexpression and patient survival

**FIGURE 2:**
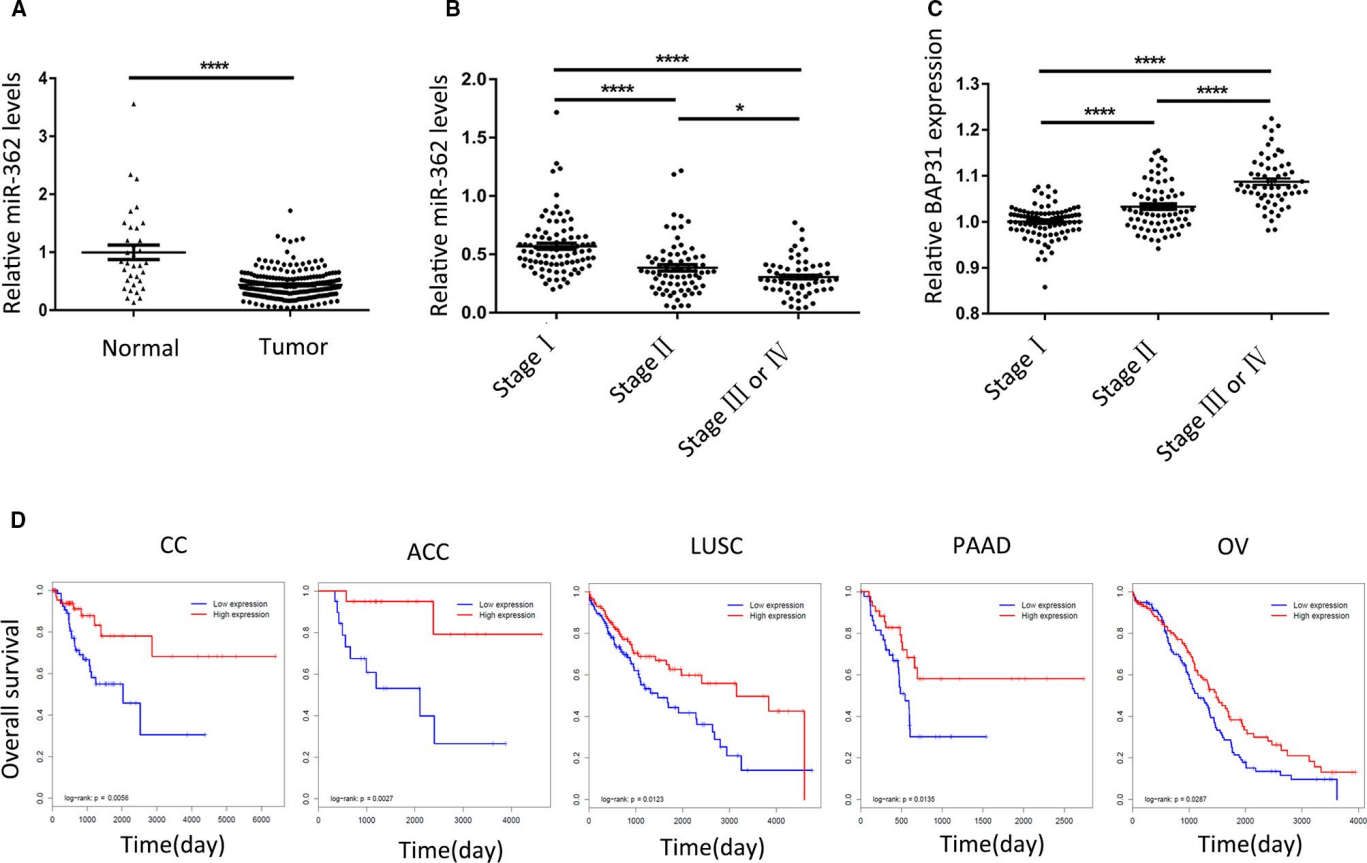
Reduced miR‐362 levels in cervical cancer associated with BAP31 protein overexpression and patient survival. (A) MiR‐362 expression was determined via qRT‐PCR in 34 paracancer tissues and 219 cancer tissues, U6 RNA was used as a control. (B) Expressions of miR‐362 in different stages of cervical cancer, for 89 patients in stage Ⅰ, 71 patients in stage Ⅱ, and 59 patients in stage Ⅲ or Ⅳ. (C) Expressions of BAP31 protein in different stages of cervical cancer, getting from the histochemistry score results after immunohistochemistry. (D) Survival curves of five different cancers with low and high miR‐362 expression from TCGA database. *p < 0.05; ****p < 0.0001

For CC, we collected 219 cancer tissues (89 patients in stage Ⅰ, 71 patients in stage Ⅱ, and 59 patients in stage Ⅲ or Ⅳ) and 34 adjacent normal tissues from our hospital and detected miR‐362 expression. MiR‐362 was revealed to be downregulated in CC tissues compared with that in normal tissues (Figure 2A). With the increase of clinical stage, the expression of miR‐362 decreased, while the expression of BAP31 increased (Figure 2B–C).

To investigate the role of miR‐362 in tumors, we studied the relationship between miR‐362 expression and patient survival. The 29 tumor types with more than 80 cases in TCGA database were analyzed, and we found that the higher expression of miR‐362, the longer survival period in at least 12 kinds of tumors, including CC (Figure 2D and Figure S1). There were significant differences among five types of tumors, which were CC, adrenocortical cancer (ACC), lung squamous cell carcinoma (LUSC), pancreatic cancer (PAAD), and ovarian cancer (OV) (Figure 2D).

### 3.3 MiR‐362 regulates the proliferation and apoptosis of CC cells by targeting BAP31

To further research on the role of miR‐362 in the development of CC by downregulating BAP31, we analyzed the effect of miR‐362 on biological behaviors of CC cells by transiently transfecting HeLa or SiHa cells with miR‐362 mimics, NC, and siBAP (as positive control).

**FIGURE 3:**
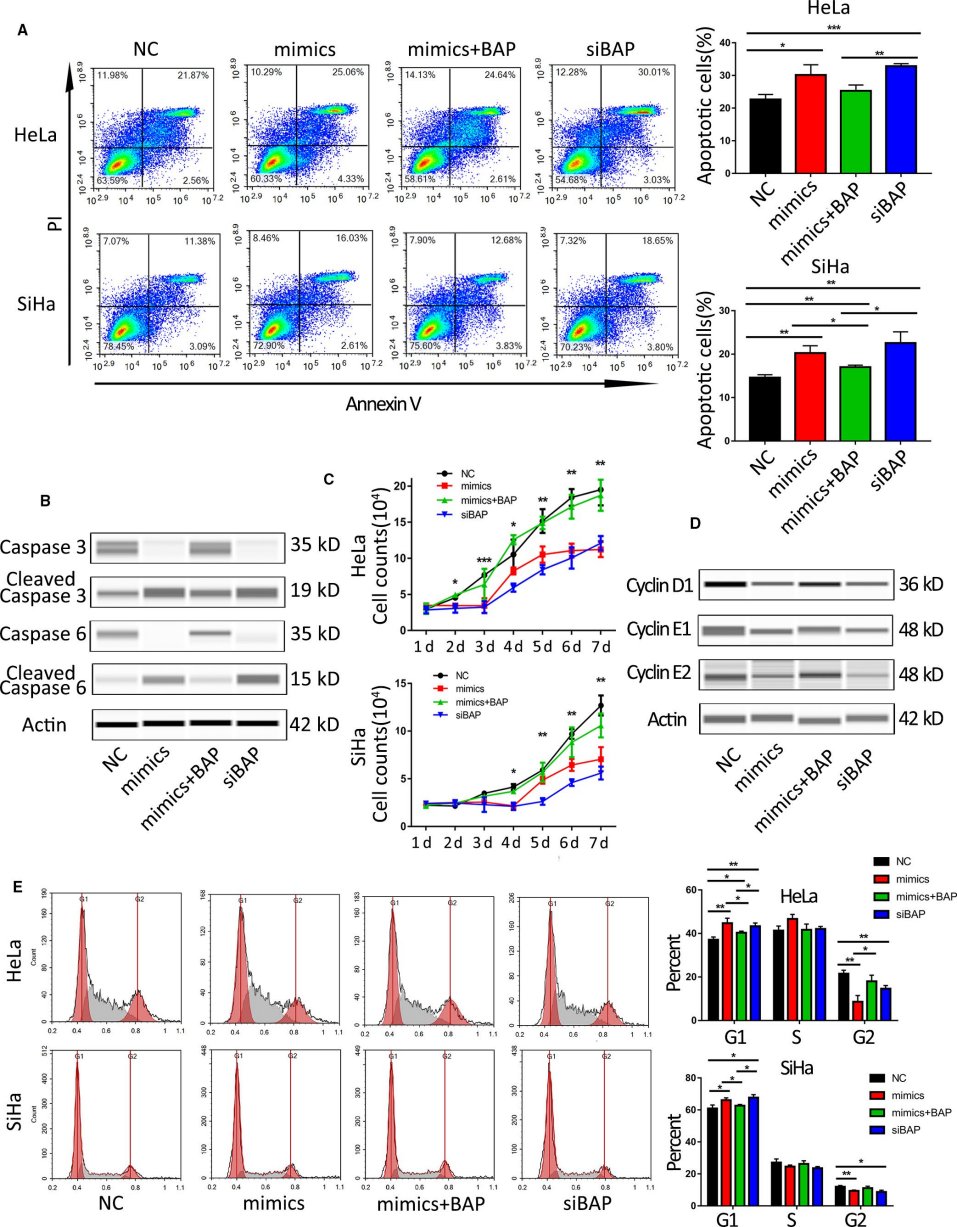
MiR‐362 regulates the proliferation and apoptosis of cervical cancer cells by targeting BAP31. (A) Apoptosis was analyzed by flow cytometry at 48 h post transfection with NC, miR‐362 mimics, miR‐362 mimics+BAP31 plasmid, and BAP31 siRNA. NC group acted as a NC and siBAP group as positive control. (B) Automated western immunoblotting analyses for caspase 3 and caspase 6 protein levels following transfection of NC, miR‐362 mimics, miR‐362 mimics+BAP31 plasmid, and BAP31 siRNA into HeLa cells. (C) The real‐time cell counting after transfection in the three groups. * showed the *p* value between mimics versus NC group. (D) Immunoblotting analyses for cyclin family protein levels following transfection in HeLa cells. (E) Flow cytometric analysis of the HeLa and SiHa cell cycle distributions with PI at 48 h post transfection with NC, mimics, miR‐362 mimics+BAP31 plasmid and siBAP. *p < 0.05; **p < 0.01; ***p < 0.001. Data are represented as the mean ± SD of three independent experiments

Cell apoptosis was observed continuously at 48 h post transfection. It was found that miR‐362 could stimulate the apoptosis of CC cells (Figure 3A). Immunoblotting results showed that caspase 3 and caspase 6 were activated after miR‐362 transfection (Figure 3B). It could be found that miR‐362 mimics inhibited cell growth by 42% and 44%, respectively, after transfection into HeLa and SiHa cells through cell counting. This effect is consistent with the knockdown of BAP31 (Figure 3C). The cell cycles were detected by flow cytometry. The proportion of cells in G0/G1 phase increased and that in G2 phase decreased after miR‐362 transfection. Immunoblotting analysis showed that the expression of cyclin family proteins was decreased after miR‐362 transfection (Figure 3D–E). This phenomenon is consistent with the results observed after knockdown of BAP31, while the antitumor effect of miR‐362 mimics was suppressed after the upregulation of BAP31 with BAP31 plasmid.

### 3.4 MiR‐362 delays CC progression in vivo

**FIGURE 4:**
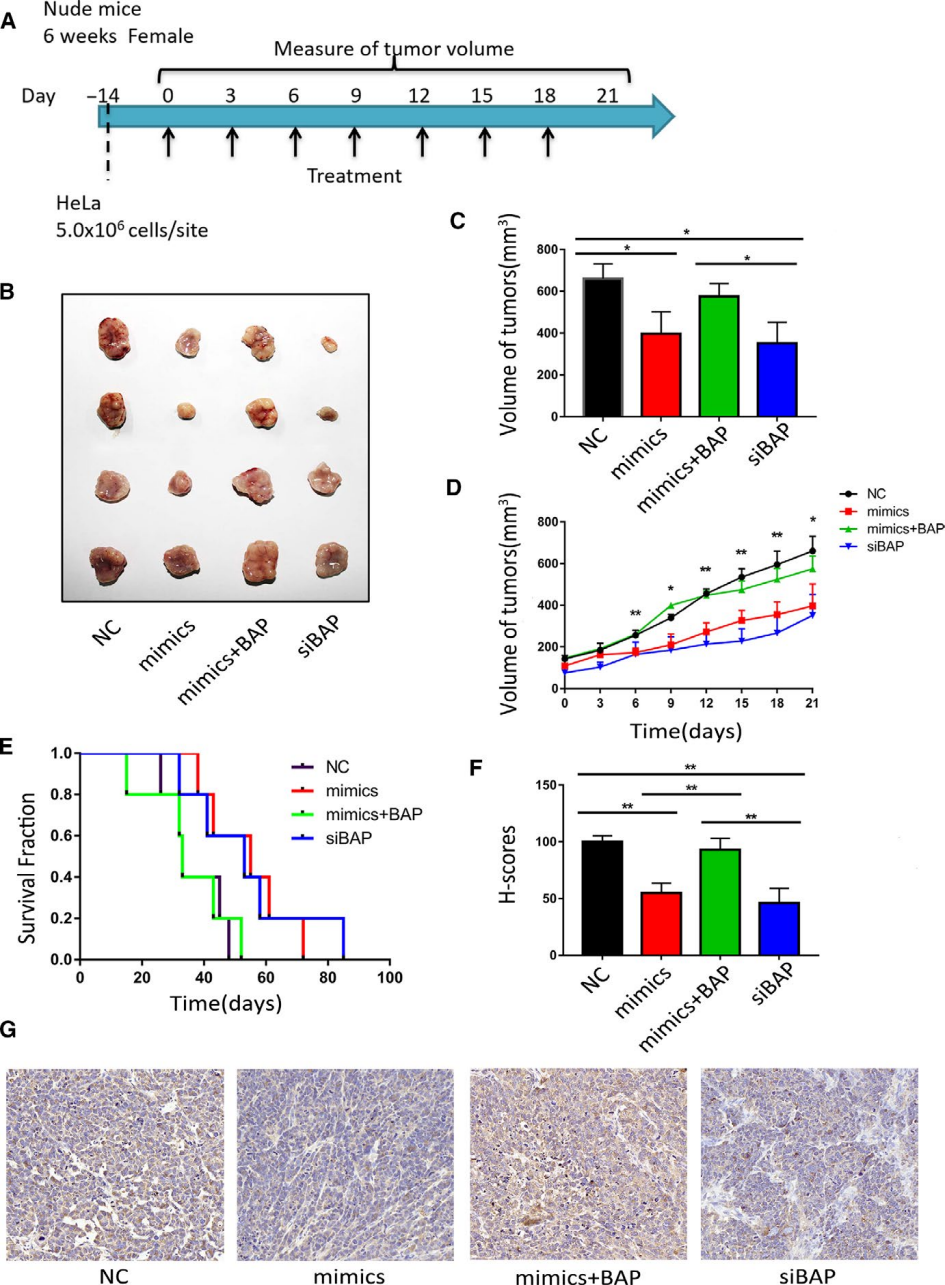
MiR‐362 delays cervical cancer progression in vivo. (A) Implementation program of tumor‐bearing nude mice experiments. (B) Image of tumors after 3 weeks’ transfection. (C) Tumor volumes were measured after 3 weeks’ transfection. (D) Growth curve of xenografts of HeLa cells transfected with NC, mimics and siBAP. * showed the *p* value between mimics versus NC. (E) Kaplan–Meier means curve representing the overall survival of the injected mice. (F and G) H‐scores (histochemistry scores) and images (20×) of BAP31 immunohistochemical staining in tumor tissues from xenograft mouse model. *p < 0.05; **p < 0.01. Data are represented as the mean ± SD of at least three independent experiments

To explore the feasibility of using miR‐362 to treat CC, we conducted an in vivo experiment using xenograft mouse model. HeLa cells were implanted into nude mice, and tumor‐bearing experiments were performed. After tumor formation, miRNA mimics were transfected to tumor cells using the living cell transfection technique (Figure 4A). The tumor size of miR‐362 treatment group was approximately 40% after 3 weeks of treatment, compared with that of NC group (Figure 4B–D). The survival time of mice was extended by 50% (Figure 4E). Immunohistochemical staining result showed that BAP31 expression in the tumor was significantly decreased after miR‐362 mimics transfection (Figure 4F–G). The antitumor effect of miR‐362 can be inhibited by BAP31 plasmid.

### 3.5 BAP31/SPTBN1 complex regulates CC cell behavior via TGFβ/Smad pathway

**FIGURE 5:**
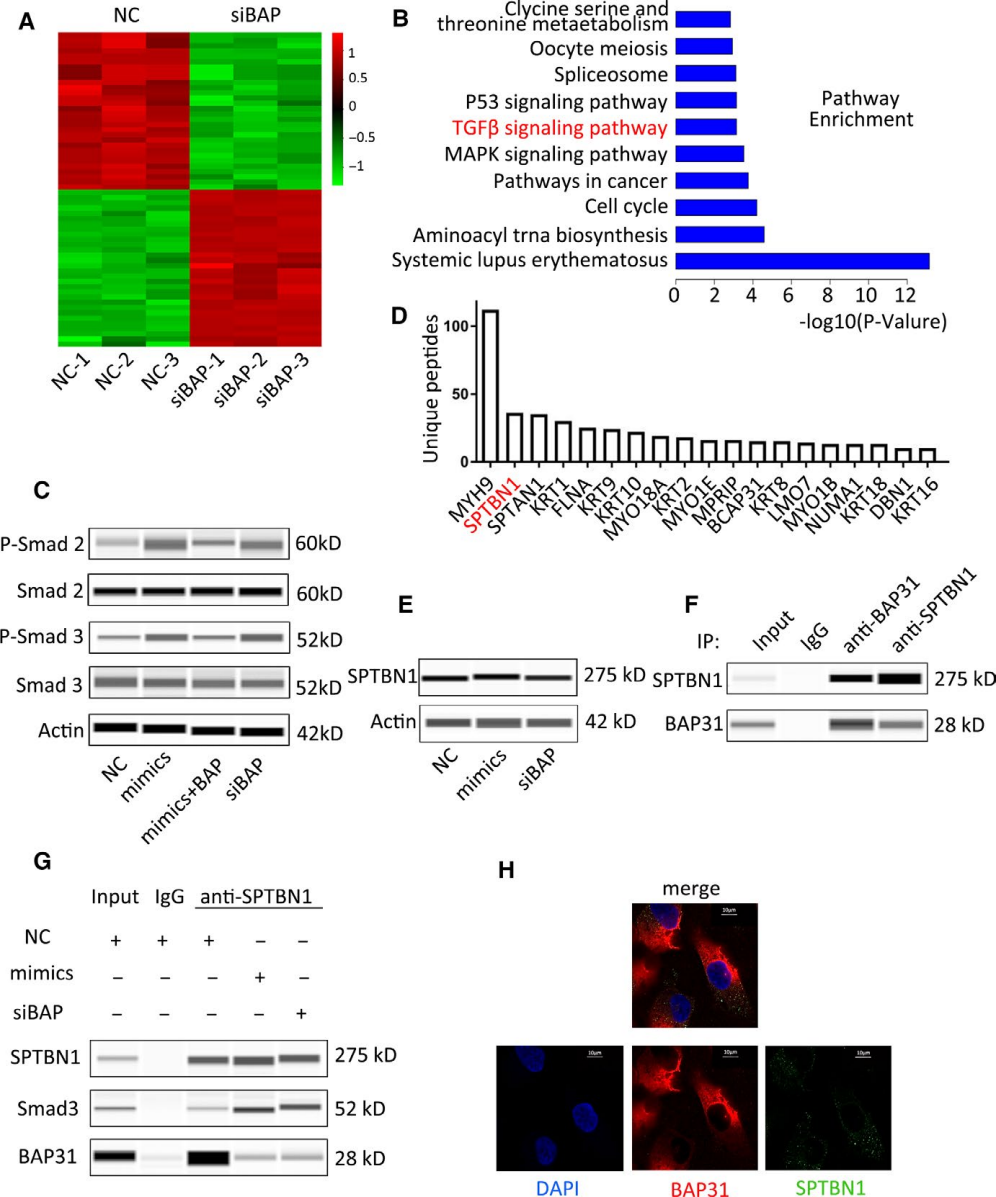
BAP31/SPTBN1 complex regulates cervical cancer cell behavior via TGFβ/Smad pathway. (A) Differentially expressed genes were identified in the HeLa wild‐type and knockout BAP31 cell by gene microarray. (B) KEGG pathway analyses of the gene chip using the IPA commercially available software. (C) Automated western immunoblotting for Smad family protein levels following transfection of NC, mimics, miR‐362 mimics+BAP31 plasmid, and siBAP into HeLa cells. (D) Genes were ranked according to their degree of correlation with BAP31 after co‐IP and MS analysis. (E) Immunoblotting analyses for SPTBN1 and BAP31 protein levels after transfection in HeLa cells. (F) Automated western immunoblotting for SPTBN1 and BAP31 protein after co‐IP with each other in HeLa cells. (G) Immunoblotting for SPTBN1, Smad 3, and BAP31 after co‐IP. (H) Immunofluorescence staining of HeLa cells with anti‐BAP31 and anti‐SPTBN1

To study the downstream mechanism by which miR‐362 regulates BAP31 in CC development, we transiently transfected the NC and siBAP into HeLa cell line, respectively. A total of 1011 differential genes were identified by gene microarray (Figure 5A). Based on the results of the gene chip, we clustered the KEGG signaling pathway. We found that the high ranking of the signaling pathways that inhibits tumor growth was TGFβ (Transforming growth factor‐β) signaling pathway (Figure 5B). Therefore, miR‐362 may play a tumor suppressive role through TGFβ pathway. Further comparing our gene chip results with the TGFβ pathway in the classical literature using the IPA commercially available software, we found that the most variable pathway in the TGF signaling pathway after knockdown of BAP31 is TGFβ/Smad pathway (Figure S3). Further detecting Smad pathway‐related proteins, the results showed that P‐Smad 2 and P‐Smad 3 were significantly increased after transfection with miR‐362 mimics or siBAP (Figure 5C). Therefore, miR‐362 and BAP31 influence TGFβ/Smad pathway activity in CC cells.

To further explore how BAP31 affects the pathway, we searched for TGFβ/Smad pathway‐associated proteins among the BAP31‐binding proteins. We conducted co‐immunoprecipitation (co‐IP) assay with anti‐BAP31 and analyzed by mass spectrometry (MS). We found that the second protein SPTBN1, which was found to be closely related to TGFβ/Smad pathway, could bind to BAP31 protein in CC cells (Figure 5D). As an adapter protein for Smad 3 in TGFβ signaling,^12^, ^13^ SPTBN1 (Spectrin beta, nonerythrocytic 1) can modulate the activation of Smad 2 and Smad 3. SPTBN1 combines with Smad 3 to trigger protein phosphorylation, which is indispensable for the activation of TGFβ/Smad signaling.^14^

Transient transfection of HeLa cells with miR‐362 mimics or siRNA revealed that downregulation of BAP31 does not affect SPTBN1 expression (Figure 5E). It was confirmed by immunoprecipitation experiments that BAP31 and SPTBN1 proteins bind to each other (Figure 5F). As SPTBN1 is an essential protein of the TGFβ/Smad pathway through binding to Smad 3, we tested whether this binding changed in miR‐362 mimics or siBAP transfection cells. The result showed that the binding between SPTBN1 and Smad 3 is more in mimics group or siBAP group, compared with that in NC group (Figure 5G). Immunofluorescence results showed that BAP31 and SPTBN1 have obvious co‐localization in HeLa cells, accounting for 57.8 ± 8.34% of SPTBN1 (Figure 5H).

**FIGURE 6:**
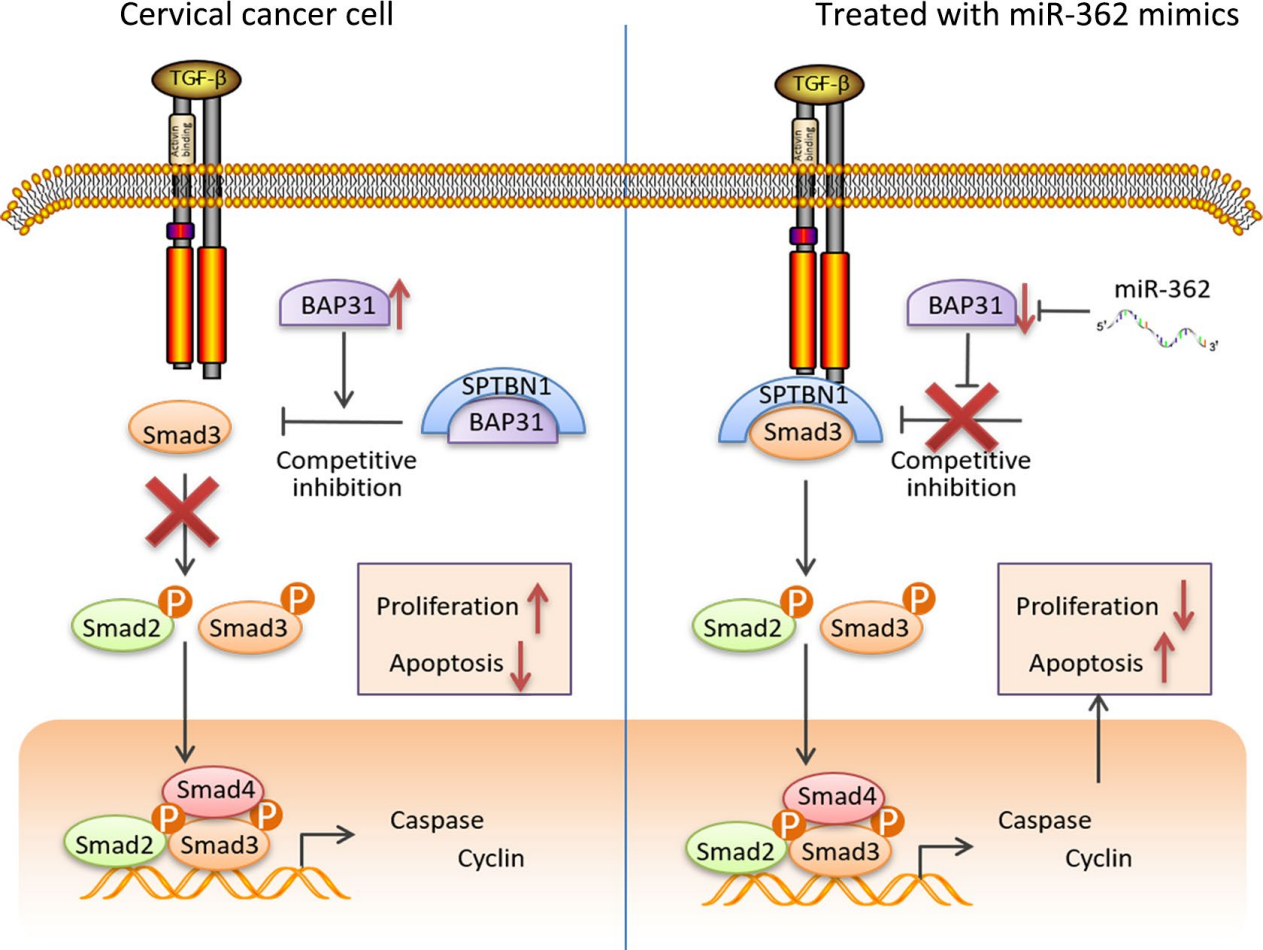
Schematic diagram of the role of miR‐362 in cervical cancer. As a negative transcriptional regulator of BAP31, abnormal low expression of miR‐362 led to increased expression of BAP31 in CC. The combination of BAP31 and SPTBN1 blocked signal transduction, and TGFβ/Smad pathway was inhibited. As a result, cervical cancer cell proliferation is accelerated, and apoptosis is reduced. In contrast, cervical cancer cells overexpressing miR‐362 via treating with miR‐362 mimics exhibited a low level of BAP31 expression. Thus, SPTBN1 could bind to Smad 3, so that TGFβ/Smad pathway is activated to inhibit cell growth

Combining above results, we obtained the possible molecular mechanism in CC. In cervical cells, miR‐362 downregulates BAP31 expression so BAP31 could not bind to SPTBN1. SPTBN1 binds to Smad 3 and the TGFβ/Smad pathway is activated to exert antitumor effect. In CC cells, miR‐362 expression decreases so that BAP31 is abnormally overexpressed. SPTBN1 is bound by BAP31 so that cannot bind to Smad 3 to activate TGFβ/Smad pathway signals. As a result, TGFβ/Smad pathway is suppressed, cell proliferation increased, and cell apoptosis decreased (Figure 6).

## 4 DISCUSSION

In this study, we showed miR‐362, as an anti‐oncomiR, inhibits CC proliferation, and promotes apoptosis. MiR‐362 downregulates BAP31 expression by directly targeting BAP31 3'‐UTR. In contrast to BAP31, miR‐362 is low expressed and negatively correlated with clinical stage in CC tissues. This miRNA regulates the proliferation and apoptosis of CC cells through directly regulating BAP31 expression in vitro and vivo. After treating with miR‐362 mimics in CC cells, miR‐362 downregulates BAP31 expression, enabling SPTBN1 to bind to Smad 3, thereby activating the TGFβ/Smad pathway and inhibiting CC progression.

BAP31 is a new tumor/testis antigen that was first screened and supposed as an oncogene by our group. In recent years, we have been committed to the exploration of the role of BAP31 in CC and other tumors. BAP31 has an immense influence on the development of CC.^3^, ^4^, ^15^ At the same time, we found that BAP31 protein is abnormally highly expressed in testes and tumors, however, BAP31 mRNA is widely expressed in most human normal tissues.^15^, ^16^ The inconsistency between this protein and mRNA strongly suggests the presence of posttranscriptional regulation. Our research screened and validated the role of miR‐362 in the regulation of BAP31 for first time, and found that miR‐362 participates in CC progression by regulating BAP31.

Recently, miRNA mimics and anti‐miRs have shown great potential acting as new anticancer agents. Modified anti‐miRs have been shown to be effective in cancer therapy.^17^, ^18^, ^19^ MRX34 is in clinical trials as a cancer treatment and also as the first miRNA mimics treatment.^20^ The use of miR‐34a in prostate cancer successfully inhibited the proliferation of tumor stem cells.^21^ In this study, on the one hand, our data suggest that the miR‐362 mimic system effectively inhibits the growth of CC cells, which indicates its potential therapeutic significance in CC. On the other hand, since miR‐362 levels are associated with a poor prognosis, miR‐362 may be a novel and useful prognostic biomarker in CC.

MiR‐362 consists of 22 bases, and its gene is located on the short chain of the X chromosome. The roles of miR‐362‐3p in different tumors are different. In gastric cancer, miR‐362‐3p is highly expressed and promotes the migration and invasion of gastric cancer cells by targeting CD82.^22^ However, in renal cell carcinoma, breast cancer, lung adenocarcinoma, and colorectal cancer, miR‐362‐3p are all under‐expressed, and inhibit various biological behaviors of cells by regulating hERG, E2F1, USF2, and PTPN.^8^, ^9^, ^23^, ^24^, ^25^ In CC, low expression of miR‐362‐3p is associated with poor prognosis,^11^ and miR‐362‐3p inhibits proliferation through MCM5 and other targets.^10^ This may be the reason why the effect of miR‐362 mimics is not weaker than siBAP on cell biological behaviors in our research results. Our study is a new supplement to the mechanism of miR‐362's antitumor effect in CC.

In our animal experiments, we used the in vivo transfection reagent for miR‐362 mimics transfection treatment, which showed a good therapeutic effect. However, CC is a solid tumor and the cells are compact. Although we perform multiple injections around the tumor for each treatment, it is still not possible for the transfection reagent to fully enter each tumor cell. If it can be combined with other cell surface‐specific markers, we believe it will have a better therapeutic effect.

As we all know, TGFβ/Smad plays an antitumor effect. When TGFβ binds to the TGFbRI‐TGFbRⅡ complex, SPTBN1 binds to Smad 3, then Smad 2 and Smad 3 are phosphorylated. Phosphorylated Smad 2 and Smad 3 form heteromeric complexes with Smad 4 and translocate into the nucleus to regulate cancer‐related genes.^13^, ^14^ It is recognized that Smad 4 plays an inhibitory role in tumors, such as breast cancer and glioma.^26^, ^27^, ^28^, ^29^ In CC cells, TGFβ has the effect of inhibiting cell proliferation and promoting apoptosis.^30^, ^31^ Studies have found that TGFβ exerts a tumor suppressive effect in the early stage of tumors, but in the later stages, its tumor suppressing effect disappears, and it even plays a tumor promoting role.^32^, ^33^ Kloth JN found that CC cell lines express high levels of TGFβ, but the reason for their inability to work is unknown.^34^ Our findings may explain the disappearance of the antitumor effect of TGFβ from a new perspective––the high expression of BAP31. The combination of BAP31 and SPTBN1 may make SPTBN1 unable to bind to Smad 3, phosphorylation cannot be performed, and the TGFβ/Smad pathway cannot be activated.

In conclusion, our data established miR‐362 as an anti‐oncomiR that regulates CC proliferation and apoptosis, and defined a set of key tumor suppressor mechanisms that can be used to overcome the progression of CC. Therefore, our results indicate that miR‐362 may be a miRNA‐based targeted therapy.

## CONFLICT OF INTEREST

The authors have no conflict of interest.

## Supporting information

FigS1‐S3‐TableS1‐S2

## Data Availability

Data available on request from the authors.
