# Supplementary material for: MiR‐362 suppresses cervical cancer progression via directly targeting BAP31 and activating TGFβ/Smad pathway
Source: Cancer Med. 2020 Nov 19;10(1):305–16. doi: 10.1002/cam4.3601 (PMC7826455; doi:10.1002/cam4.3601)
Supplement: Supplementary file 1 — FigS1‐S3‐TableS1‐S2 [file CAM4-10-305-s001.docx]

**Supplementary Material**

**MiR-362 suppresses cervical cancer progression via directly targeting BAP31 and activating TGFβ/Smad pathway**

**Supplementary Figure 1.**
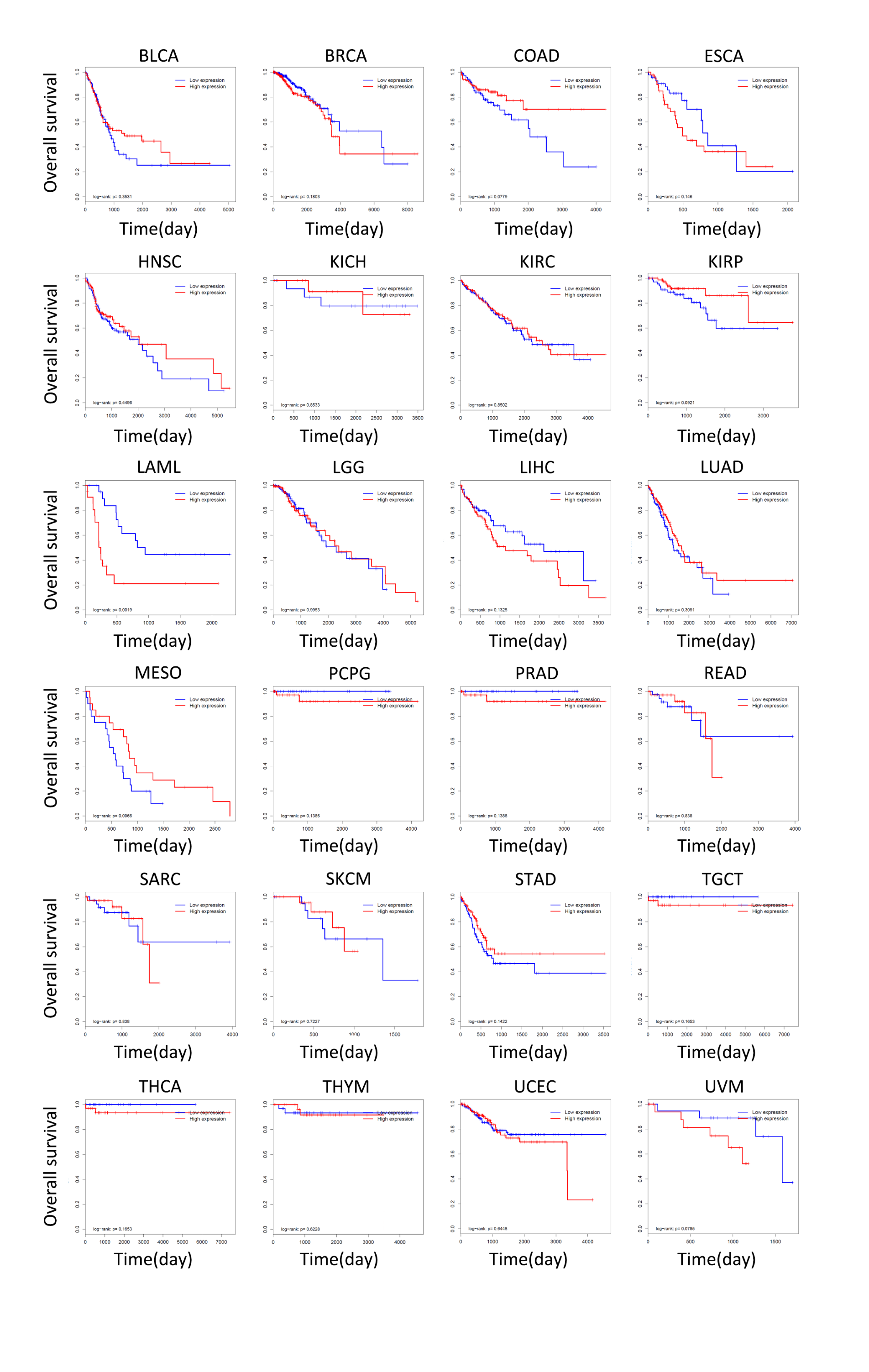


**Supplementary Figure 1. Survival curves of different cancers with low and high miR-362 expression from TCGA database.Supplementary Figure 2.**


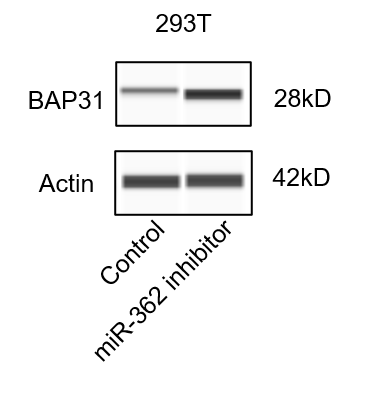


**Supplementary Figure 2. MiR-362 inhibitor up-regulates the expression of BAP31 in 293T cells.**

**Supplementary Figure 3.**


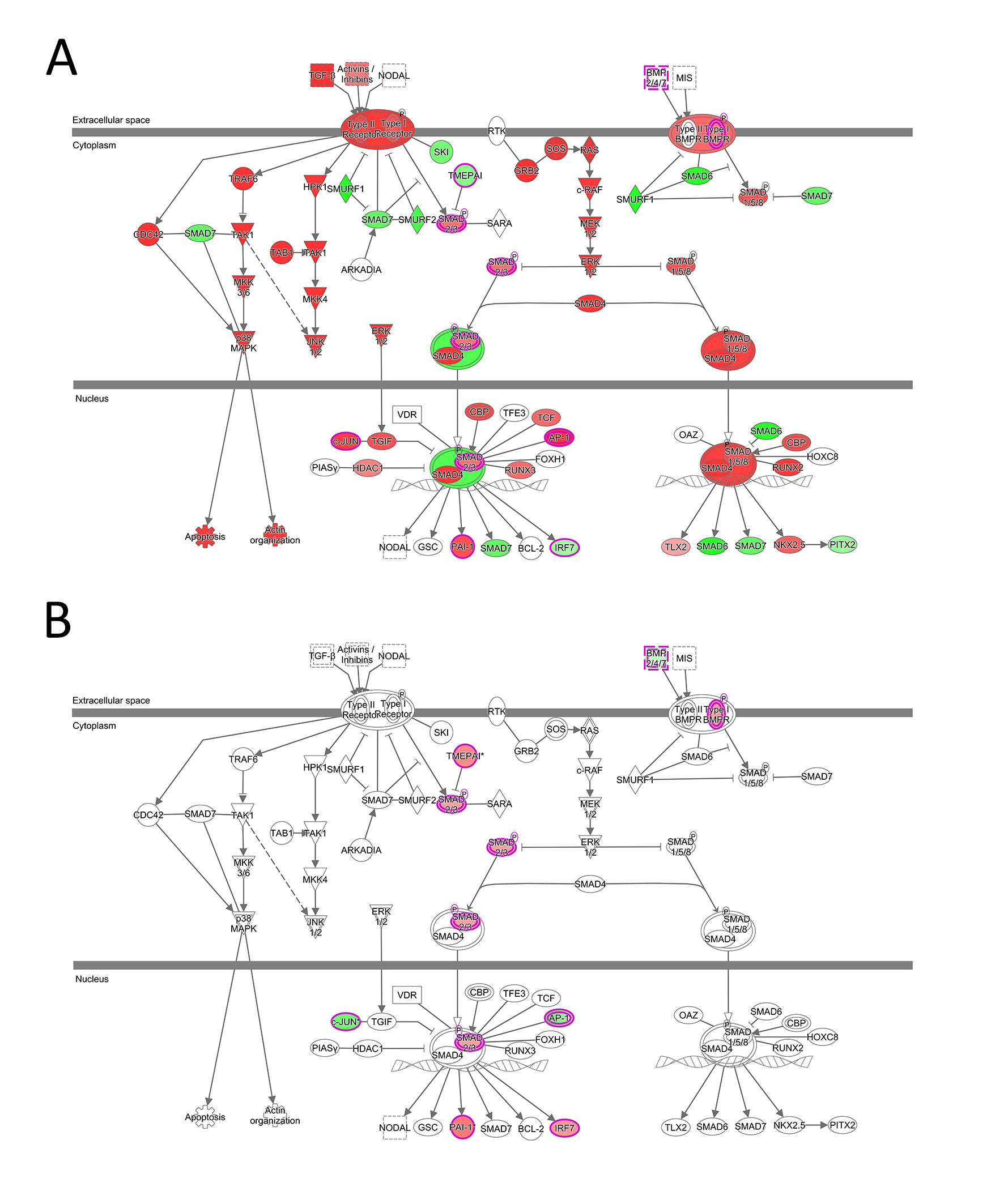


**Supplementary Figure 3. The most variable pathway in the** **TGFβ signaling pathway after knockdown of BAP31 is TGFβ /Smad pathway using the IPA commercially available software.**

**A** Canonical pathway map supported by literature TGF-β signaling from IPA software. Red represents up-regulation of gene expression, green represents down-regulation, and shades of color indicate degree of change. **B** Map of our gene chip results versus classic TGF-β pathway from IPA software.

**Supplementary Table S1. Sequence used for transient transfection of cells**

| Gene name |  | Sequences |
| --- | --- | --- |
| Negative control | Sense： | 5'-UUC UCC GAA CGU GUC ACG UTT-3' |
|  | Antisense： | 5'-ACG UGA CAC GUU CGG AGA ATT-3' |
| miR-143-3p mimics | Sense： | 5'-UGA GAU GAA GCA CUG UAG CUC-3' |
|  | Antisense： | 5'-GCU ACA GUG CUU CAU CUC AUU-3' |
| miR-195-5p mimics | Sense： | 5'-UAG CAG CAC AGA AAU AUU GGC-3' |
|  | Antisense： | 5'-CAA UAU UUC UGU GCU GCU AUU-3' |
| miR-214-3p mimics | Sense： | 5'-ACA GCA GGC ACA GAC AGG CAG U-3' |
|  | Antisense： | 5'-UGC CUG UCU GUG CCU GCU GUU U-3' |
| miR-218-5p mimics | Sense： | 5'-UUG UGC UUG AUC UAA CCA UGU-3' |
|  | Antisense： | 5'-AUG GUU AGA UCA AGC ACA AAU-3' |
| miR-362-3p mimics | Sense | 5'-AAC ACA CCU AUU CAA GGA UUC A-3' |
|  | Antisense： | 5'-AAU CCU UGA AUA GGU GUG UUU U-3' |
| miR-424-5p mimics | Sense： | 5'-CAG CAG CAA UUC AUG UUU UGA A-3' |
|  | Antisense： | 5'-CAA AAC AUG AAU UGC UGC UGU U-3' |
| miR-497-5p mimics | Sense： | 5'-CAG CAG CAC ACU GUG GUU UGU-3' |
|  | Antisense： | 5'-AAA CCA CAG UGU GCU GCU GUU-3' |
| Control (for inhibitor) |  | 5'-CAG UAC UUU UGU GUA GUA CAA-3' |
| miR-362-3p inhibitor |  | 5'-UGA AUC CUU GAA UAG GUG UGU U-3' |
| BAP31 siRNA | Sense： | 5'-GGU GAA CCU CCA GAA CAA UTT-3' |
|  | Antisense： | 5'-AUU GUU CUG GAG GUU CAC CTT-3' |

**Supplementary Table S2. Sequences of Primers for Real Time PCR Used in the Experiment**

| Gene name | Sequences |
| --- | --- |
| miR-143-3p | F: 5'- TGT GAC ACT GAG ATG AAG CAC TG-3' |
|  | R: 5'- TAT GGT TTT GAC GAC TGT GTG AT-3' |
| miR-195-5p | F: 5'-TCA TCA CAT AGC AGC ACA GAA AT-3' |
|  | R: 5'-TAT GGT TTT GAC GAC TGT GTG AT-3' |
| miR-214-3p | F: 5'-CCA ACA GCA GGC ACA GAC A-3' |
|  | R: 5'-TAT GGT TTT GAC GAC TGT GTG AT-3' |
| miR-218-5p | F: 5'-CGA TTC CAT TTG TGC TTG ATC T-3' |
|  | R: 5'-TAT GGT TTT GAC GAC TGT GTG AT-3' |
| miR-362-3p | F: 5'-GCC GAA ACA CAC CTA TTC AAG-3' |
|  | R: 5'-TAT GGT TTT GAC GAC TGT GTG AT-3' |
| miR-424-5p | F: 5'-GCC AGC AGC AAT TCA TGT-3' |
|  | R: 5'-TAT GGT TTT GAC GAC TGT GTG AT-3' |
| miR-497-5p | F: 5'-GCA AAC AGC AGC ACA CTG TG-3' |
|  | R: 5'-TAT GGT TTT GAC GAC TGT GTG AT-3' |
| U6 | F: 5'-ATT GGA ACG ATA CAG AGA AGA TT-3' |
|  | R: 5'-GGA ACG CTT CAC GAA TTT G-3' |
